# Phylogenetic Analysis of MERS-CoV in a Camel Abattoir, Saudi Arabia, 2016–2018

**DOI:** 10.3201/eid2612.191094

**Published:** 2020-12

**Authors:** Maged Gomaa Hemida, Daniel K.W. Chu, Yen Y. Chor, Samuel M.S. Cheng, Leo L.M. Poon, Abdelmohsen Alnaeem, Malik Peiris

**Affiliations:** Kafrelsheikh University, Kafrelsheikh, Egypt (M. Hemida); King Faisal University, Al-Hasa, Saudi Arabia (M. Hemida, A. Alnaeem);; The University of Hong Kong (D.K.W. Chu, Y.Y. Chor, S.M.S. Cheng, L.L.M. Poon, M. Peiris)

**Keywords:** MERS, coronavirus, dromedary, camels, Saudi Arabia, phylogeny, abattoir, respiratory infections, zoonoses, viruses, MERS-CoV, Middle East respiratory syndrome

## Abstract

We detected Middle East respiratory syndrome coronavirus (MERS-CoV) RNA in 305/1,131 (27%) camels tested at an abattoir in Al Hasa, Eastern Province, Saudi Arabia, during January 2016–March 2018. We characterized 48 full-length MERS-CoV genomes and noted the viruses clustered in MERS-CoV lineage 5 clade B.

Middle East respiratory syndrome (MERS) coronavirus (MERS-CoV) is a zoonotic disease of concern for global public health (*1*,*2*). Dromedary camels are the source of zoonotic infection (*3*). During 2016–2018, a total of 80 full-length MERS-CoV genome sequences were available from human infections in the Arabian Peninsula where all zoonotic disease has occurred, but only 30 sequences from dromedary camels were available, highlighting the need for contemporary dromedary MERS-CoV sequence data.

During November 2015–June 2018, nasal and rectal swab specimens were collected, typically on a monthly basis, from dromedary camels slaughtered at an abattoir and camel market complex in Al Hasa, Eastern Province, Saudi Arabia (Appendix). Most camels for slaughter were bred locally, but some camels were imported from Somalia or Sudan for slaughter. Imported camels came through the port of Jeddah, usually via a large central camel market in Riyadh.

Nasal and rectal swab specimens were collected from 1,131 camels; 4–143 camels were sampled each month. Overall, 288 (25.5%) nasal and 85 (7.5%) rectal swabs were MERS-CoV–positive as confirmed by reverse transcription PCR (RT-PCR; Appendix); cycle threshold values ranged from 15.3 to 39.1 (median 33.6). Most (68/85; 80%) positive rectal swab specimens were collected from animals that also had a positive nasal swab. Overall, 305 (27%) camels sampled were MERS-CoV–positive from either nasal or rectal swabs. Despite regular exposure to infected camels, none of the abattoir workers had diagnosed clinical MERS disease.

MERS-CoV–positive samples were detected during most months in which samples were tested. Age, sex, date of sampling, and breed data were available for 847 camels. Among animals for which age and sex data were available, RT-PCR positive rates for MERS-CoV were not statistically significantly different by age or sex. Among local camels, MERS-CoV–positive rates by breed were 81/227 (35.7%) Magaheem, 19/87 (21.8%) Sofor, and 27/158 (17.1%) Wodaoh. Among imported camels, 21/146 (14.4%) from Somalia and 64/221 (29%) from Sudan were MERS-CoV–positive.

We obtained 48 full genomes of MERS-CoV from the camel samples; dates of sampling were available for 35 (GenBank accession nos. MN654970–5017). We did not detect evidence of deletions in accessory or other genes. Our newly generated virus genomes phylogenetically clustered within the recombinant lineage 5 clade, a novel recombinant clade that has become progressively dominant in Saudi Arabia since 2014 (*4*,*5*) (Appendix Figure). The 48 sequences in this study appear to cluster into 2 groups, which we named group A and group B for ease of description rather than a formal taxonomic designation (Figure). Other sublineages within lineage 5 appear to have gone extinct with no human or animal viruses detected since 2016. Virus group A had viruses sampled from 2014–2017, whereas group B had viruses sampled in 2014–2018. Both virus groups cocirculated in the region during the study period (Figure).

**Figure:**
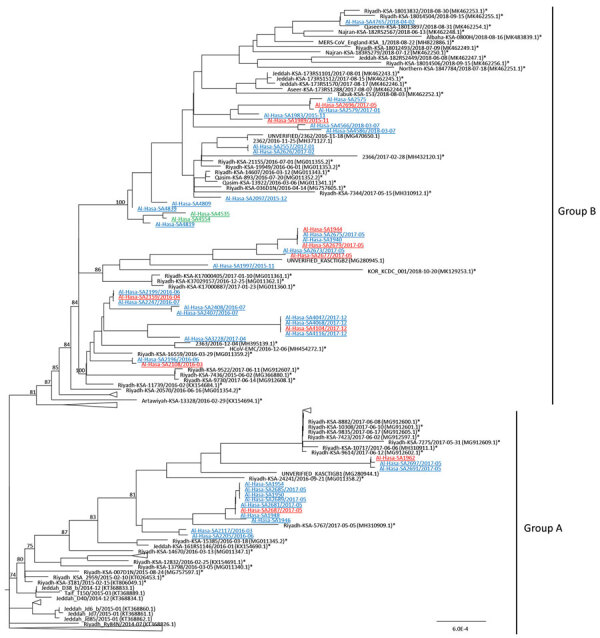
Phylogeny of Middle East respiratory syndrome coronavirus (MERS-CoV) sequenced from nasal and rectal samples collected from camels in an abattoir, Saudi Arabia. Phylogeny was constructed by using IQTREE (http://www.iqtree.org) with the automatic nucleotide transition model selection. Branch supports, shown at major nodes, were generated by ultrafast bootstrap approximation (Appendix). Genomes generated from this study are underlined; asterisks (*) indicate viruses from humans. Blue indicates viruses from camels from Saudi Arabia; red indicates viruses in camels imported from Sudan; green indicates viruses in camels imported from Somalia. The overall topology of the phylogeny of MERS-CoV also is available (Appendix Figure). Scale bar indicates 10^4^ mutations per site.

Genetically identical viruses were collected mostly during the same sampling period, suggesting cross-infection in the market. However, identical viruses sometimes were from samples collected 1 month apart, such as SA2557 and SA2626, or 3 months apart, such as SA2199, SA2159, and SA2247, suggesting reintroduction of viruses from the same herd or area into the abattoir at different times. Although we sampled imported camels from Somalia and Sudan, the viruses we detected were clade B lineage 5 viruses rather than the clade C viruses that are known to be enzootic in Africa (*6*). In several instances, viruses from camels from Sudan (for example, SA4104/2017 in December 2017 or SA2687/2017 in May 2017) were almost identical to viruses concurrently detected in camels from Saudi Arabia, indicating likely cross-infection in the camel market. Virus cross-infection and amplification in the camel market could explain the high overall MERS-CoV–positive rate in the abattoir.

A virus from a patient from Saudi Arabia who had diagnosed MERS in the United Kingdom in August 2018 (MERS-CoV_England-KSA_1/2018-08-22) was found to be closely genetically related (99.81% similarity) to a camel virus sampled during this study in 2018 (Figure). As previously reported, viruses from camels and humans interleave within the phylogenetic tree (*7*), suggesting that viruses in camels continue to be the source of human infections through separate zoonotic transmission events without sublineage separation between viruses in camels and humans.

In conclusion, our study suggests multiple lineage 5 clade B viruses continue to be dominant among camels in eastern Saudi Arabia. Camels imported from Sudan and Somalia also had evidence of MERS-CoV B lineage 5 clade viruses prevalent in the Arabian Peninsula, rather than clade C viruses known to be enzootic in camels in Africa. These data suggest imported camels likely acquired MERS-CoV after arriving in Saudi Arabia and that lineage 5 viruses have the greater evolutionary fitness and appear to outcompete other viral lineages, which is concordant with other recently reported data (*8*). The high rates of MERS-CoV we detected and viral phylogeny suggest likely cross-transmission of MERS-CoV within the camel market and abattoir complex, even among imported animals.

Appendix Additional information on methods for sequencing Middle East respiratory syndrome coronavirus from camels in an abattoir, Saudi Arabia.
