# Supplementary material for: Phylogenetic Analysis of MERS-CoV in a Camel Abattoir, Saudi Arabia, 2016–2018
Source: Emerg Infect Dis. 2020 Dec;26(12):3089–91. doi: 10.3201/eid2612.191094 (PMC7706958; doi:10.3201/eid2612.191094)
Supplement: Appendix — Additional information on methods for sequencing Middle East respiratory syndrome coronavirus from camels in an abattoir, Saudi Arabia. [file 19-1094-Techapp-s1.pdf]

Article DOI: https://doi.org/10.3201/eid2612.191094

Phylogenetic Analysis of MERS-CoV in a Camel Abattoir, Saudi Arabia, 2016–2018

Appendix

Additional Methods

Nasal and rectal swabs were collected, typically on a monthly basis, from dromedary camels slaughtered at an abattoir in Al Hasa, Eastern Province, Saudi Arabia during November 2015–May 2018. Samples were collected according to the animal ethics protocols of the National Committee of Bio-Ethics, King Abdul-Aziz City of Science and Technology, by Royal Decree no. M/59.

Camels for slaughter were sourced from the adjacent large market-complex, which covers 2.3 km^2 and is divided between sheep and camel pens, auction areas, the slaughterhouse, and animal feed barns. The complex holds ≈5,000 camels at any time in >600 camel pens, each of which holds a few to >40 animals. Camels traded at the market include those for slaughter for meat, milk production, personal collections, genetic stock improvements, and for Mezaeen, camel beauty contests. Most camels in the market are of breeds local to Saudi Arabia and largely are sourced from the eastern and central regions of the country. Imported camels for slaughter came from Somalia or Sudan and came through the port of Jeddah, usually through a large central camel market in Riyadh. Camels remain in the market for a few days to several months. Given proximity and movement of fomites and persons, ample opportunities for cross-transmission of Middle East respiratory syndrome coronavirus (MERS-CoV) between camel pens exists. Each day, 20–40 camels are slaughtered at the abattoir; the numbers vary by seasons and demand. Abattoir workers were male, do not use masks or gloves, but sometimes wear boots and aprons.

Methods used for specimen collection, virus nucleic acid extraction, quantitative reverse transcription-PCR (qRT-PCR) for virus RNA detection, upE gene confirmed by open reading

frame 1a (ORF1a) gene targets, were described previously (*1*,*2*). Samples with upE qRT-PCR cycle threshold (Ct) values <30 were subjected to virus whole-genome sequencing by generating cDNA with gene specific primers and RT-PCR for 2–4 kb overlapping virus genome regions, as previously described (*1*). PCR products of the genomes were sequenced by using the Solexa (Illumina, https://www.illumina.com) sequencing platform with Nextera (Illumina) library preparation method. Virus genomes were assembled with ≥100 folds of sequencing coverage.

All available human and camel MERS-CoV genome sequences from the Middle East and Arabian Peninsula (n = 459) and representative sequences from Africa >25.6 kb (85% of the full genome, n = 482) were downloaded from Genbank (https://www.ncbi.nlm.nih.gov/genbank) for phylogenetic analysis. Sequences were aligned using MAFFT (https://mafft.cbrc.jp/alignment/software) and phylogeny was constructed by using IQTREE (http://www.iqtree.org) with the automatic nucleotide transition model selection and ultrafast bootstrap approximation.

References

1. Chu DKW, Hui KPY, Perera RAPM, Miguel E, Niemeyer D, Zhao J, et al. MERS coronaviruses from camels in Africa exhibit region-dependent genetic diversity. Proc Natl Acad Sci U S A. 2018;115:3144–9. PubMed https://doi.org/10.1073/pnas.1718769115

2. Corman VM, Eckerle I, Bleicker T, Zaki A, Landt O, Eschbach-Bludau M, et al. Detection of a novel human coronavirus by real-time reverse-transcription polymerase chain reaction. Euro Surveill. 2012;17:20285. PubMed https://doi.org/10.2807/ese.17.39.20285-en

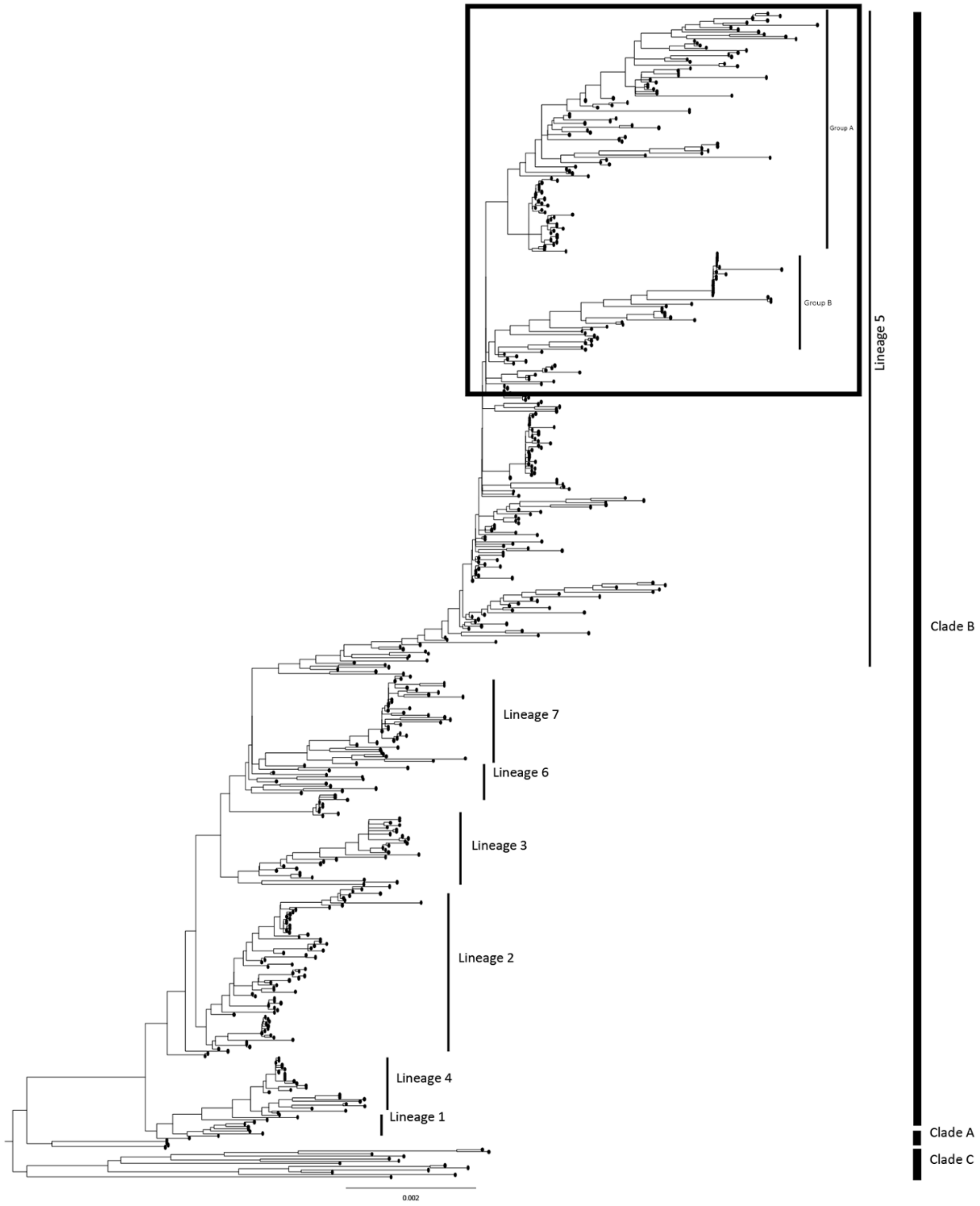

Appendix Figure. Phylogenetic tree of Middle East respiratory syndrome coronavirus (MERS-CoV) detected in humans and camels. Clades and lineages of MERS-CoV are indicated. The area within the box encompasses viruses sequenced in this study. Phylogeny was constructed using IQTREE (http://www.iqtree.org) with the automatic nucleotide transition model selection.
